# Time serial transcriptome reveals *Cyp2c29* as a key gene in hepatocellular carcinoma development

**DOI:** 10.20892/j.issn.2095-3941.2019.0335

**Published:** 2020-05-15

**Authors:** Qi Wang, Qin Tang, Lijun Zhao, Qiong Zhang, Yuxin Wu, Hui Hu, Lanlan Liu, Xiang Liu, Yanhong Zhu, Anyuan Guo, Xiangliang Yang

**Affiliations:** ^1^College of Life Science and Technology, Huazhong University of Science and Technology, Wuhan 430074, China; ^2^Experimental Transplantation and Immunology Branch, National Cancer Institute, National Institutes of Health, Bethesda, MD 20892, USA

**Keywords:** Cyp2c29, hepatocellular carcinoma, NF-κB, proliferation, time series gene expression

## Abstract

**Objective:** Hepatocellular carcinoma (HCC) is a severely lethal cancer that usually originates from chronic liver injury and inflammation. Although progress on diagnosis and treatment is obvious, the cause of HCC remains unclear. In this study, we sought to determine key genes in HCC development.

**Methods:** To identify key regulators during HCC progression, we performed transcriptome sequencing to obtain time series gene expression data from a mouse model with diethylnitrosamine-induced liver tumors and further verified gene expression and function *in vitro* and *in vivo*.

**Results:** Among the differentially expressed genes, *Cyp2c29* was continuously downregulated during HCC progression. Overexpression of Cyp2c29 suppressed NF-κB activation and proinflammatory cytokine production by increasing the production of 14,15-epoxyeicosatrienoic acid *in vitro*. Furthermore, overexpression of Cyp2c29 *in vivo* protected against liver inflammation in mouse models of liver injury induced by both acetaminophen and CCl_4_. Two human homologs of mouse *Cyp2c29*, *CYP2C8* and *CYP2C9*, were found to be downregulated in human HCC progression, and their expression was positively correlated with overall survival in patients with HCC (significance: *P* = 0.046 and 0.0097, respectively).

**Conclusions:** Collectively, through systematic analysis and verification, we determined that *Cyp2c29* is a novel gene involved in liver injury and inflammation, which may be a potential biomarker for HCC prevention and prognosis determination.

## Introduction

Hepatocellular carcinoma (HCC) is the sixth most frequently diagnosed cancer and the second most common cause of cancer-related death worldwide^1,2^. Although the pathophysiology of HCC is not clearly understood, HCC typically arises in chronically injured and inflamed livers. Persistent injury is characterized by inflammatory cell infiltration accompanied by cytokine production^3,4^. Chronic inflammation triggers compensatory cell proliferation and causes genetic mutations that may induce carcinogenesis^5,6^. The identification of key molecules and mechanisms involved in liver injury in HCC will be important for HCC prevention and diagnosis.

CYP enzymes are xenobiotic-metabolizing enzymes that are primarily found in the liver cells of rodents and humans^7–9^. These enzymes are involved in eicosanoid metabolism and regulate a diverse set of homeostatic and inflammatory processes. The expression levels of CYP genes are closely associated with the progression of liver diseases. For example, *Cyp2c29*, *Cyp2c50*, *Cyp2c55* and *Cyp2j5* transcription levels have been found to be suppressed in a mouse model of nonalcoholic steatohepatitis^10^. In addition, decreased expression of CYP genes, such as *CYP2C8*, *CYP2C9*, and *CYP2C19*, has been observed in patients with HCC^11–13^. However, the role of CYP genes in the progression of HCC is not well characterized.

High-throughput sequencing combined with bioinformatics analysis provides a systematic way to explore crucial genes and mechanisms involved in complex diseases^14^. Previously, we investigated miRNA and transcription factor (TF) coregulatory networks to reveal regulators in blood diseases^15–17^. Our findings indicated that complex regulatory relationships involved in disease progression can be illuminated by transcriptome and network analysis.

In the present study, we detected differentially expressed genes (DEGs) during the progression of liver injury by performing time series gene expression analysis. We focused on *Cyp2c29*, which was found to be significantly downregulated in a mouse model of liver injury. Our results demonstrated that *Cyp2c29* may serve as a new biomarker for the prevention, diagnosis and prognostic determination of HCC.

## Materials and methods

### Mice

Four-week-old male C57BL/6 mice were purchased from the Hubei Research Center of Laboratory Animals [Wuhan, China; quality certification number: SCXK (E) 2015-0018]. The animal studies were approved by the Ethics Committee on Animal Experimentation of Huazhong University of Science and Technology (permit number: S2264) and followed the guidelines of the Science and Technology Department of Hubei Province (China). All mice were treated humanely and were maintained in individual ventilated cages, given autoclaved water, and fed irradiated food.

### Diethylnitrosamine (DEN)-induced liver injury model

To mimic the process of HCC development, mice were orally administered DEN (TCI, Japan) at a dose of 0.16 mmol·kg^−1^ body weight once per week for the first 10 weeks and fed with a high fat diet (HFD) for 30 weeks^18,19^. In the control group, mice were administered sesame oil (the solvent for DEN) once per week for the first 10 weeks and fed a basal chow diet. At weeks 0, 10, 20, 25, and 30, mice were sacrificed, and liver tissues were collected for histological examination and RNA extraction.

### Histological examination

Liver tissues were fixed in 4% formaldehyde for 24 h. Paraffin sections (5 μm) were prepared for hematoxylin and eosin (H&E) staining for general morphological observation. Masson trichrome staining was used to reveal collagen fibers. The sections were observed under a light microscope (Nikon Eclipse TE2000-U) and then photographed at 100×, 200×, or 400× magnification. Liver histology scoring was performed as described previously^20^.

### Sequencing and identification of differentially expressed genes

High-throughput RNA-seq techniques were used to detect gene expression. Three biological samples per group were sequenced. For RNA-seq, ribosomal RNA was first removed, and 150 bp paired-end sequencing was carried out on the Illumina HiSeq 3000 platform. All RNA isolation, sequencing, and data filtering procedures were performed by RiboBio (Guangzhou, China). Assembly, alignment, quantification, and profiling of the RNA-seq data were performed on our own server. First, the quality of the reads was verified with FastQC (version: 0.11.3). Then HISAT2 (version: 2.0.5) was used to map the sequencing reads to the mouse genome (GRCm38). Subsequently, StringTie (version: 1.2.2) was used to assemble the alignments into potential transcripts according to the reference sequences^21^. Finally, transcript abundance was calculated as the FPKM. Additionally, NOISeq was used to identify DEGs with a false discovery rate threshold < 0.01 and an |FC| ≥ 1.5. The data have been deposited at BIG Data Center (http://bigd.big.ac.cn) under accession number CRA000931.

### Selection of key genes and pathways

For functional annotation, DEG enrichment analysis was performed with the Database for Annotation, Visualization and Integrated Discovery (DAVID; https://david.ncifcrf.gov/tools.jsp). The Gene Ontology (GO) terms and Kyoto Encyclopedia of Genes and Genomes (KEGG) pathways are displayed in bubble plots. The rich factor in the bubble plots was calculated as the ratio of the number of genes that mapped to a certain pathway to the total number of genes in that pathway. Gene expression patterns were identified by hierarchical clustering and are displayed with a heat map. Furthermore, we investigated the most important pathways (Benjamini-Hochberg adjusted *P* < 0.05) and their crosstalk. For key gene selection, genes in the top-ranked pathways (*P* < 0.01) were deemed important. Then, we focused on the genes expressed in more than 80% of the groups. PLS-DA was used to screen the important genes that contributed the most to differentiation of the groups. The cutoff was set as a variable importance in projection (VIP) score > 2.0 (the VIP score is a weighted sum of squares of the PLS loadings)^22^.

### *Cyp2c29* plasmid transfection

The *Cyp2c29* pENTER-C-GFP plasmid and plasmid-harboring *E. coli* were supplied by Vigene Bioscience. The plasmid was sequenced for validation (GenBank accession number NM_007815.3). Then plasmids were extracted from *E. coli* with an EndoFree Plasmid Kit (Tiangen, China). Purified plasmids were transfected into HL-7702 cells according to the Lipofectamine^®^ 3000 Transfection Reagent (Thermo Fisher, USA) protocol. Hydrodynamics-based transfection of *Cyp2c29* plasmids into the mouse liver was performed as described previously^23^.

### Treatment of HL-7702 cells with epoxyeicosatrienoic acid (EET)

The hepatocyte line HL-7702 was transfected with *Cyp2c29* plasmid or treated with exogenous 11,12-EET or 14,15-EET (Cayman, USA) at a concentration of 100 nmol/L^24^. Cell viability was assessed with a Cell Counting Kit-8 (Dojindo, Japan). The levels of 14,15-EET hydrolysate (14,15-DHET), a stable metabolite of 14,15-EET, in the cell supernatant were measured with an ELISA kit (Detroit R&D, USA) according to the manual.

### Isolation of hepatocytes and non-parenchymal cells

Purification of primary hepatocytes was performed as described previously^25^. Hepatocytes were separated with 90% Percoll solution^26^, and non-parenchymal cells were separated with 20% iodixanol solution^27^.

### Incubation of primary mouse hepatocytes with LPS-activated RAW264.7 cell supernatant

RAW264.7 cells were activated with 200 ng/mL LPS (Sigma-Aldrich, USA) with or without 100 nmol/L 14,15-EET added to the medium^24^. The supernatant was collected 12 h later. Cytokines in the supernatant were quantified with a LEGENDplex™ Mouse Inflammaton Panel (BioLegend, USA). A series of RAW264.7 supernatants with volume ratios ranging from 1/100 to 1/10 were mixed into the primary mouse hepatocyte medium. Cell viability was assessed with a Cell Counting Kit-8 after 24 h of incubation.

### APAP- and CCl_*4*_-induced liver injury models

Acetaminophen (APAP)- and carbon tetrachloride CCl_4_)-induced mouse liver injury models were established by intraperitoneal injection of mice with 500 mg/kg APAP and 10 mL/kg CCl_4_ (0.2%, dissolved in olive oil), respectively. The mice were divided into 3 groups per experiment: a control group (*n* = 6), a model group (100 μg of empty plasmid diluted to 0.1 mL/g body weight), and a *Cyp2c29* group (100 μg of *Cyp2c29* plasmid diluted to 0.1 mL/g body weight). Forty-eight hours after plasmid injection, APAP and CCl_4_ were administered to the *Cyp2c29* group and model group. Mice were sacrificed at 6 h and 24 h after APAP administration (or 24 h and 48 h after CCl_4_ administration) for collection of sera and liver tissues. Serum aminotransferase (ALT) and aspartate aminotransferase (AST) levels were determined with an automatic biochemical analyzer (Beckman, Germany). Hepatocyte proliferation was measured with Ki67 staining. The data are expressed as the mean ± standard error of the mean. RNA extraction and high-throughput sequencing were performed by BGI (Wuhan, China). Gene expression, DEGs, and gene enrichment were analyzed with the methods described above for the time series gene expression analysis. For the TF regulatory network analysis, we used a method described in our previous publication^28^. Finally, Cytoscape (version 3.2.1) was used to visualize the networks. The data have been deposited at BIG Data Center (http://bigd.big.ac.cn) under and accession number CRA000931.

### Statistical analysis

In the APAP and the CCl_4_ experiments, Bonferroni and Dunnett T3 tests, respectively, were used for multiple comparisons after ANOVA based on homoscedasticity. A significance level of 0.05 and a power of 0.9 were used to estimate the necessary sample size. On the basis of our experimental design and pre-experiments, the difference between the means divided by the pooled standard deviation (effect size) was greater than 3; *n* = 3.370694 was obtained for each group with the pwr.t.test function in R programming language. Ultimately, a sample size of 6 for each group was selected to ensure the robustness of our experiments. Statistical analysis was performed in IBM SPSS Statistics (version 20). Statistical significance was determined by Student’s t test for the other experiments.

### qRT-PCR

Total RNA from hepatic tissues or cells was extracted with TRIzol reagent (Thermo Fisher, USA). RNA was reverse-transcribed into cDNA with a reverse transcription kit (Takara, Japan), and qRT-PCR was performed with a SYBR^®^ Premix Ex Taq II Kit (Takara, Japan) and a 7500 Real-Time PCR System (Applied Biosystems). The GAPDH gene was selected as the housekeeping gene for normalization. Primer sequences are shown in **Supplementary Table S1**.

### Western blot analysis

To enable loading of equal amounts, protein concentrations were determined with a bicinchoninic acid protein assay reagent kit (Beyotime, China). The protein samples were adjusted to the same concentration before loading. HL-7702 cell and liver tissue protein extracts were fractionated by electrophoresis on 12% SDS-polyacrylamide gels (Beyotime, China) and then transferred to nitrocellulose membranes. The protein blots were blocked with 5% BSA dissolved in Tris-buffered saline with Tween 20 and then incubated with appropriate primary antibodies against NF-κB p65 (1:500; Proteintech, 10745-1-AP), p-NF-κB p65 (1:500; Cell Signaling Technology, #3031), cyclin D1 (1:500; Cell Signaling Technology, #2978), IKK (1:1000; Proteintech, 15649-1-AP), phospho-IKK (1:500; Cell Signaling Technology, #2694), cleaved caspase-3 (1:1000; Cell Signaling Technology, #9664), GAPDH (1:1000; Proteintech, 60004-1-1g), and β-actin (1:2000; Cell Signaling Technology #8457) at 4 °C for 12 h. The membranes were then washed 3 times and incubated with a 1:3000 dilution of horseradish peroxidase-conjugated secondary antibody (Yeasen, China) for 2 h. Then, the proteins were detected with an enhanced chemiluminescence system (Bio-Rad, USA).

### Targeted proteomics

Quantification of liver Cyp2c29 protein in mice after APAP and CCl_4_ treatment was performed with targeted proteomics according to the previous reports^29^. Tissue samples were lysed in a solution containing 200 μL of thiourea buffer (50 mM Tris-Cl, pH 8, 7 M urea, 2 M thiourea, and 1× protease inhibitor cocktail), 800 μL of ice-cold acetone, and 10 mM dithiothreitol. After centrifugation at 13,000 × g for 20 min at 4 °C, the precipitated pellets were washed 3 times and collected. The dried pellets were dissolved in 200 μL of thiourea buffer for further analysis. A triple TOF 5600+ LC-MS/MS system (AB SCIEX) was used for targeted MS analysis with parallel reaction monitoring. Spectrum library generation was analyzed in ProteinPilot software. The peptides were selected for quantification according to the ion signals in the spectrum library.

### Survival analysis in human HCC

To verify the effects of *Cyp2c29* homologous genes in humans, we investigated the effects of homologous gene expression on clinical survival. The homologous genes were obtained from HomoloGene (https://www.ncbi.nlm.nih.gov/homologene/), and their similarities and identities were calculated with Needle from EMBOSS (version: 6.6.0). The gene expression data for 363 patients with clinical survival information were downloaded from The Cancer Genome Atlas (TCGA)^30^. Kaplan-Meier analysis and log-rank tests were used for survival analysis as in GSCALite (http://bioinfo.life.hust.edu.cn/web/GSCALite/)^31^. The samples were divided into high and low expression groups for each gene according to the median expression in the 363 patients. A value of *P* < 0.05 was considered significant. Subsequently, the patients were divided into 4 groups according to their clinical information (by TNM stage: I, II, III, and IV), and the CYP gene expression at different stages is shown in a boxplot. The normality of the distribution was examined, and significance was tested with the Kruskal-Wallis test.

## Results

### Transcriptomic changes during liver injury in a DEN-induced HCC mouse model

**Figure 1:**
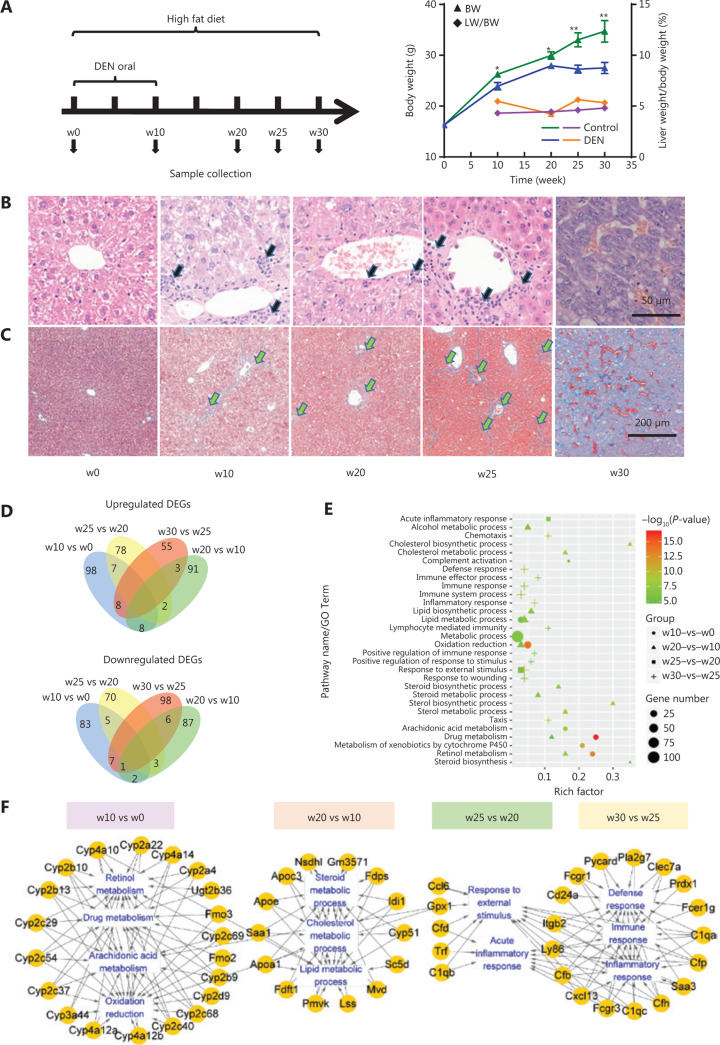
Experimental design, biological characteristics, and time series gene expression data for the DEN-induced liver injury model. (A) Experimental scheme and body weight and liver weight changes. (B) H&E staining and (C) Masson staining of liver tissues at weeks 0, 10, 20, and 25, and tumor tissues at week 30. Black arrow: inflammatory infiltration. Green arrow: fibrosis. (D) Number of up- and downregulated DEGs in different comparisons. The upper Venn diagram displays the upregulated DEGs, whereas the lower Venn diagram displays the downregulated DEGs. (E) Functional annotation of the DEGs. (F) Gene-based pathway crosstalk analysis for liver DEGs in the DEN-induced mouse model. *P < 0.05; **P < 0.01.

To mimic the process of hepatocarcinogenesis, we used low doses of DEN and HFD to establish a mouse model of liver injury. Liver tissues were collected at weeks 0, 10, 20, 25, and 30 (w0, w10, w20, w25, and w30). Mice showed body weight loss, hepatocyte necrosis, and inflammatory infiltration from w10 onward when they were given DEN and fed the HFD (**Figure 1A, B**). At w25, severe liver fibrosis was detected in the DEN treatment group, as confirmed by the observation of extensive collagen deposition and bridges between vessels after Masson trichrome staining (**Figure 1C**). At w30, tumors were observed in 5 out of 6 mice in the DEN treatment group. In addition, H&E staining of tumor tissues showed a typical trabecular growth pattern (**Figure 1B**).

To identify key regulators in HCC progression, liver samples were collected at 5 time points with 3 replicates per time point, and RNA-seq was performed (**Figure 1A**). From all samples, we obtained a total of 16,195 expressed genes [fragments per kilobase of transcript per million mapped reads (FPKM) > 0]. Genes with FPKM values > 20 (approximately 10% of the genes) were considered highly expressed. The 5 groups shared 403 highly expressed genes (**Supplementary Figure S1A**), which were mainly involved in carboxylic acid, protein, and triglyceride metabolic processes, and the acute inflammatory response (**Supplementary Figure S1B**).

Gene expression was analyzed to identify key regulators, and 564 DEGs were identified through comparisons between every 2 adjacent time points (**Figure 1D**). Functional enrichment analysis revealed that most of the DEGs in the w10 *vs.* w0 comparison were enriched in drug metabolism, retinol metabolism, and oxidation reduction functions (**Figure 1E**), thus indicating metabolic dysfunction in the liver, whereas DEGs in the w20 *vs.* w10 comparison were mainly enriched in lipid metabolic and biosynthetic pathway functions, such as those involving cholesterols and steroids. The prominent processes associated with DEGs in the w25 *vs.* w20 comparison were complement activation and inflammatory responses (**Figure 1E**). Next, we examined the key genes involved in the most significant pathways for each comparison. Through gene-pathway crosstalk analysis, we found that CYP family genes acted as important nodes connecting multiple pathways, including drug metabolism, oxidation reduction, arachidonic acid metabolism, and retinol metabolism pathways (**Figure 1F**). The importance of CYP genes in these pathways has been investigated in several studies^32–34^, and our findings were consistent with the published results.

### *Cyp2c29* is a potential key gene consistently downregulated in HCC development, as confirmed through transcriptome analysis and expression validation

**Figure 2:**
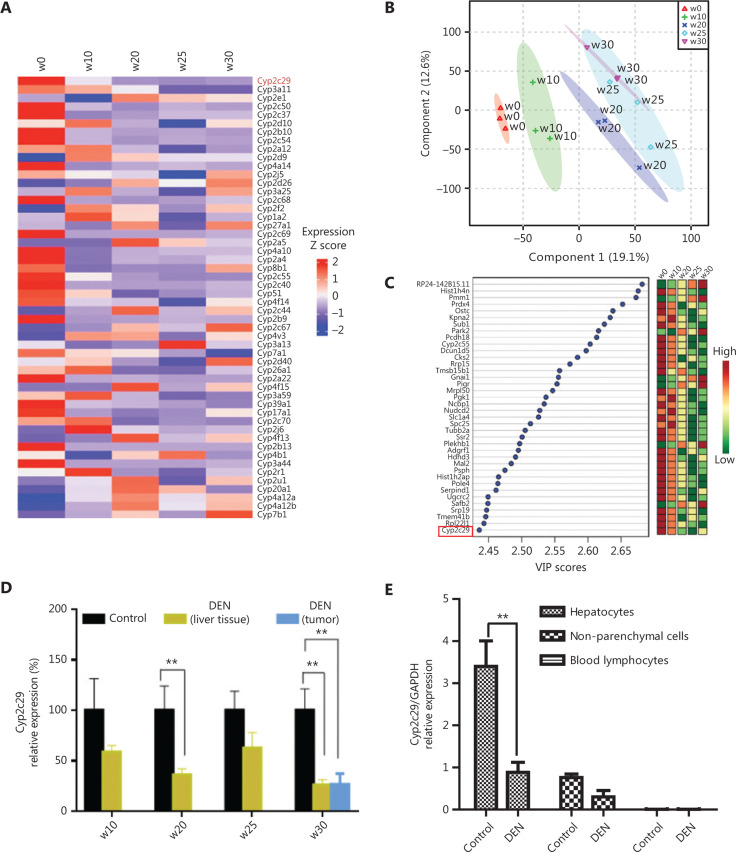
Identification of *Cyp2c29* as a gene with significantly altered expression in DEN-induced liver injury. (A) Expression changes and clustering of CYP family genes. (B) Important genes identified by PLS-DA and (C) relevant VIP scores. (D) Validation of decreased *Cyp2c29* expression by qRT-PCR at different time points of DEN-induced liver injury (*n* = 6). (E) *Cyp2c29* expression in different liver cells subjected to injury induced by DEN (24 h after 100 mg/kg intraperitoneal injection). **P < 0.01.

To further analyze the key genes involved in HCC progression, we screened all 52 CYP genes detected at least one time point that met a threshold of FPKM > 5.0 (**Figure 2A**). Approximately half the CYP genes were continuously downregulated from w0 to w30. This finding is highly relevant, given the reported findings that P450 genes are downregulated during the inflammatory response^11–13^. Most of the downregulated genes belonged to the *Cyp2b* and *Cyp2c* subfamilies. Among the identified genes, *Cyp2c29* was highly expressed at w0 but decreased during the progression of liver injury (fold change (FC) = −2.88 from w0 to w10, FC = −3.02 from w10 to w20).

In addition, we performed partial least squares discriminant analysis (PLS-DA) of all the genes to identify key genes at each time point. A total of 419 marker genes were obtained with VIP scores > 2.0 that contributed to distinguishing the 5 groups (**Figure 2B**). Furthermore, the DEGs with VIP scores in the top 10% were determined (**Figure 2C**), and *Cyp2c29* was again identified as a core gene. The decreased expression of the *Cyp2c29* gene during liver injury was further confirmed by qRT-PCR analysis (*n* = 6) (**Figure 2D**). The expression of *Cyp2c29* was lower in the DEN-treated group than in the control group at the same time point. Hepatocytes constitute approximately 60%–80% of the total cell population in the liver^35^. We isolated different cells hepatocytes, non-parenchymal cells and blood lymphocytes in the liver and found that the expression of *Cyp2c29* was mainly reduced in the hepatocytes in livers with injury induced by DEN (24 h after 100 mg/kg intraperitoneal injection, *P* < 0.01) (**Figure 2E**).

### *Cyp2c29* suppresses NF-κB activation and inflammation-stimulated cell proliferation

**Figure 3:**
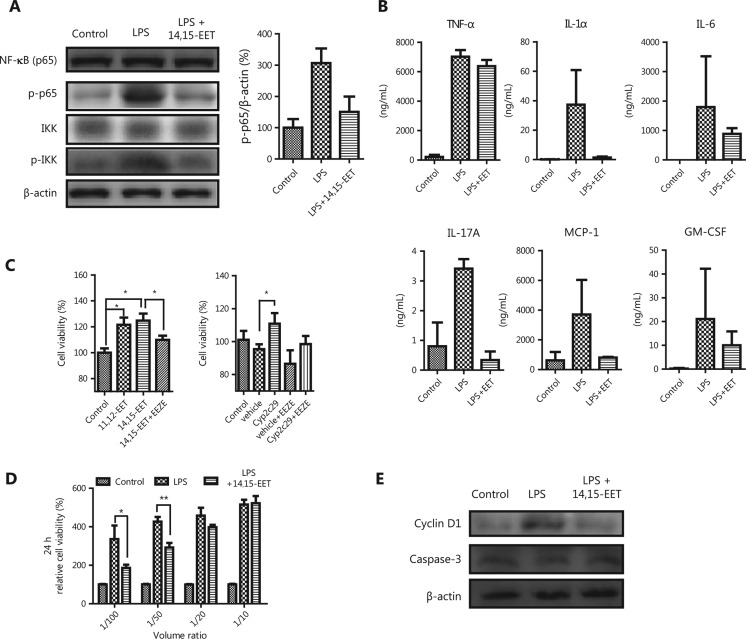
*Cyp2c29* suppressed NF-κB activation and inflammation-stimulated hepatocyte proliferation. (A) Western blot analysis of NF-κB (p65), p-p65, and p-IKK expression in RAW264.7 cells. (B) Expression of inflammatory cytokines in the supernatant of macrophage RAW264.7 activated by LPS with or without 14,15-EET treatment. (C) Cell viability of *Cyp2c29*-transfected or EET-treated HL-7702 cells. (D) Cell viability of primary mouse hepatocytes incubated with different volume ratios of supernatant from different groups of RAW264.7 cells. (E) Western blot analysis of cyclin D1 and caspase-3 expression in primary mouse hepatocytes. *P < 0.05; **P < 0.01.

As a member of the Cyp2c family, Cyp2c29 is an endothelial EET synthase^36^. EETs have anti-inflammatory activity and limit inflammation damage to blood vessels^37^. To investigate the role of EET in HCC development, we evaluated the effects of EET on hepatocytes under an inflamed environment. To clarify the influence of inflammatory mediators, we treated RAW264.7 cells with lipopolysaccharide (LPS) to mimic an inflamed environment*.* EET decreased the protein levels of p-NF-κB (p65) in LPS treated RAW264.7 cells (**Figure 3A**). Moreover, the high levels of inflammatory cytokines in the supernatant (IL-1α, IL-6, IL-17A, and MCP-1) secreted by LPS-activated RAW264.7 cells decreased after EET treatment (**Supplementary Figure S3** and **Figure 3B**). The supernatant from LPS-treated RAW264.7 cells significantly enhanced the viability of primary mouse hepatocytes, whereas decreases in viability were observed after treatment with a combination treatment with EET (**Figure 3D**), thus suggesting that Cyp2c29 may suppress cell growth by attenuating inflammation.

We further investigated the role of Cyp2c29 under a normal environment. The liver cell line HL-7702 was transfected with a* Cyp2c29* expression plasmid. qRT-PCR results confirmed Cyp2c29 expression (**Supplementary Figure S2**), which was further verified by EET production analysis. Significantly enhanced 14,15-DHET levels were observed in the supernatant of the Cyp2c29-overexpressing HL-7702 cells (**Supplementary Figure S2C**). HL-7702 cells overexpressing Cyp2c29 or treated with exogenous EETs showed significantly greater cell proliferation compared with that of the control (**Figure 3C**), and this effect was blocked by the EET antagonist 14,15-epoxyeicosa-5(*Z*)-enoic acid (**Figure 3C**). Our results were consistent with those from a report indicating that EET enhances cell proliferation *in vitro*^38^. The data suggested that EET plays different roles in cell proliferation in the presence or absence of inflammatory conditions. Western blot analysis showed that the expression of cyclin D1, a critical regulator of cell cycle progression and cell proliferation, was diminished in the EET and LPS co-treatment group, but caspase-3 expression was not significantly changed (**Figure 3E**). Similar results were observed in the hepatocellular carcinoma H22 cell line (**Supplementary Figure S4**).

### Overexpression of *Cyp2c29* decreases inflammation in liver injury models

**Figure 4:**
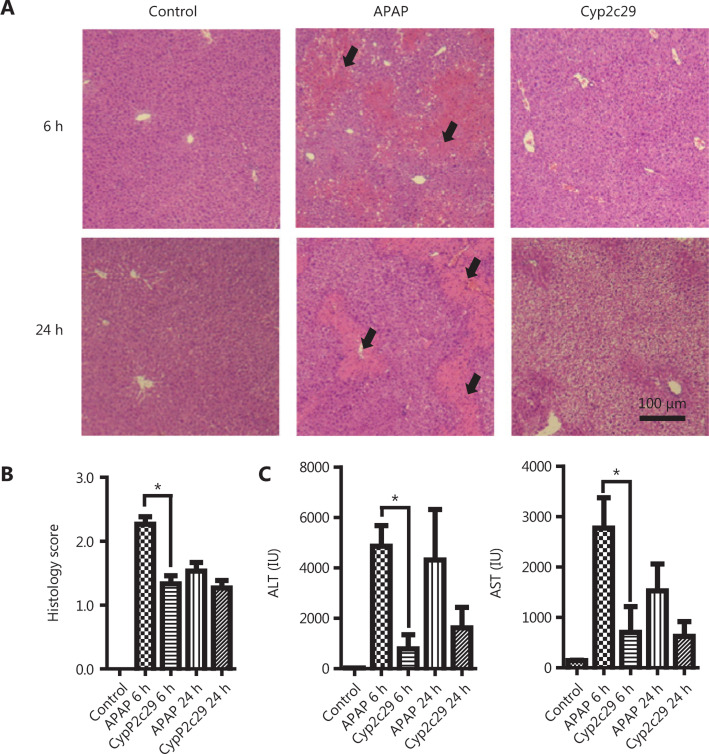
Overexpression of *Cyp2c29* decreased inflammation in the APAP-induced liver injury model. The mice in the *Cyp2c29* group received 100 μg of *Cyp2c29* plasmid DNA 48 h before APAP administration, whereas those in the APAP group received an equivalent empty vector 48 h before APAP administration. The control group did not receive APAP or plasmid DNA. (A) Liver H&E staining and (B) histology scores of APAP-induced liver injury. Black arrows: necrotic areas. (C) ALT and AST levels at 6 h and 24 h after APAP administration. *P < 0.05.

NF-κB is a master regulator of inflammation and is also known to be a central signaling node between hepatic injury and HCC^39^. In agreement with results from previous reports, we found that the NF-κB signaling pathway was activated during liver inflammation, on the basis of data from DEN-induced HCC in mice (**Supplementary Figure S5**). To verify the role of *Cyp2c29* in liver injury, we used 2 other liver injury models induced by APAP and CCl_4_. These models are characterized by typical NF-κB activation and inflammatory responses^40,41^. A *Cyp2c29* expression plasmid was transfected into mice, which were then injected with APAP or CCl_4_ to induce liver injury. The animals were sacrificed at 6 h and 24 h after APAP administration. *Cyp2c29* expression was increased at both the mRNA and protein levels in the APAP-treated group transfected with the* Cyp2c29* plasmid (**Supplementary Figure S6**). Pathological examination indicated that the cell necrosis area was diminished in the *Cyp2c29* group, as revealed by H&E staining of liver tissue (**Figure 4A, B**). Elevated ALT and AST levels were observed in the APAP group treated with empty vector plasmid (**Figure 4C**), whereas significantly diminished ALT and AST levels were detected in the *Cyp2c29* group (APAP group *vs.* *Cyp2c29* group at 6 h, *P* < 0.05). These results illustrated the protective effect of *Cyp2c29* during liver injury. CCl_4_-treated mice also exhibited significantly lower *Cyp2c29* expression than control mice (**Supplementary Figure S6**), thus suggesting that *Cyp2c29* is consistently downregulated during liver injury. Less inflammatory infiltration and lower ALT levels were observed in the *Cyp2c29* group (transfected with *Cyp2c29* plasmid and treated with CCl_4_) than in the CCl_4_ model group (**Supplementary Figure S7A–D**). In addition, the number of CD68-positive cells in the liver was smaller in the *Cyp2c29* group than the CCl_4_ model group (**Supplementary Figure S7E**), possibly because of decreased inflammatory infiltration and liver injury^42^.

### Transcriptome analysis and *in vivo* experiments reveal a potential anti-inflammatory function of *Cyp2c29* in liver injury

**Figure 5:**
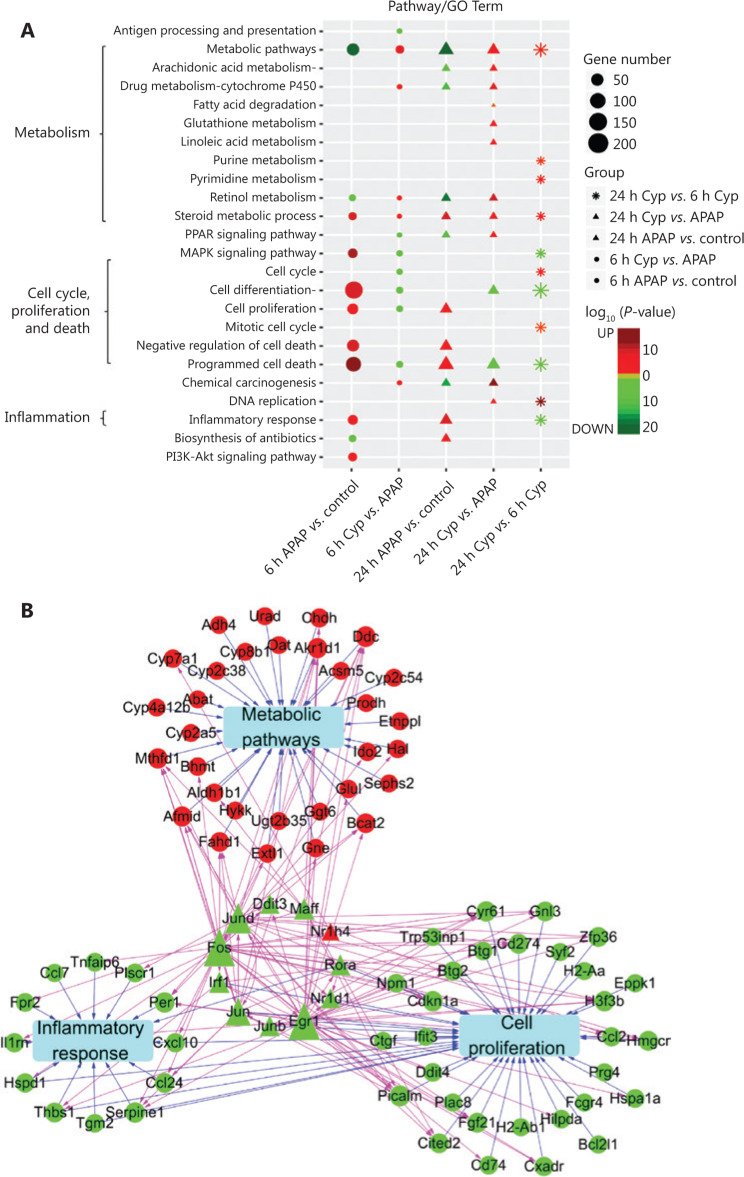
Transcriptome analysis revealed the potential functions of *Cyp2c29* in APAP-induced liver injury. (A) GO functional enrichment of DEGs from the APAP (model) group *vs.* control group, and *Cyp2c29* group *vs.* APAP group comparisons at 6 h and 24 h after APAP treatment. Red: upregulated. Green: downregulated. (B) TF regulation network and crosstalk for the 6 h *Cyp2c29* group *vs.* APAP group comparison. Red: upregulated. Green: downregulated. Diamonds: TFs. Circles: genes.

**Figure 6:**
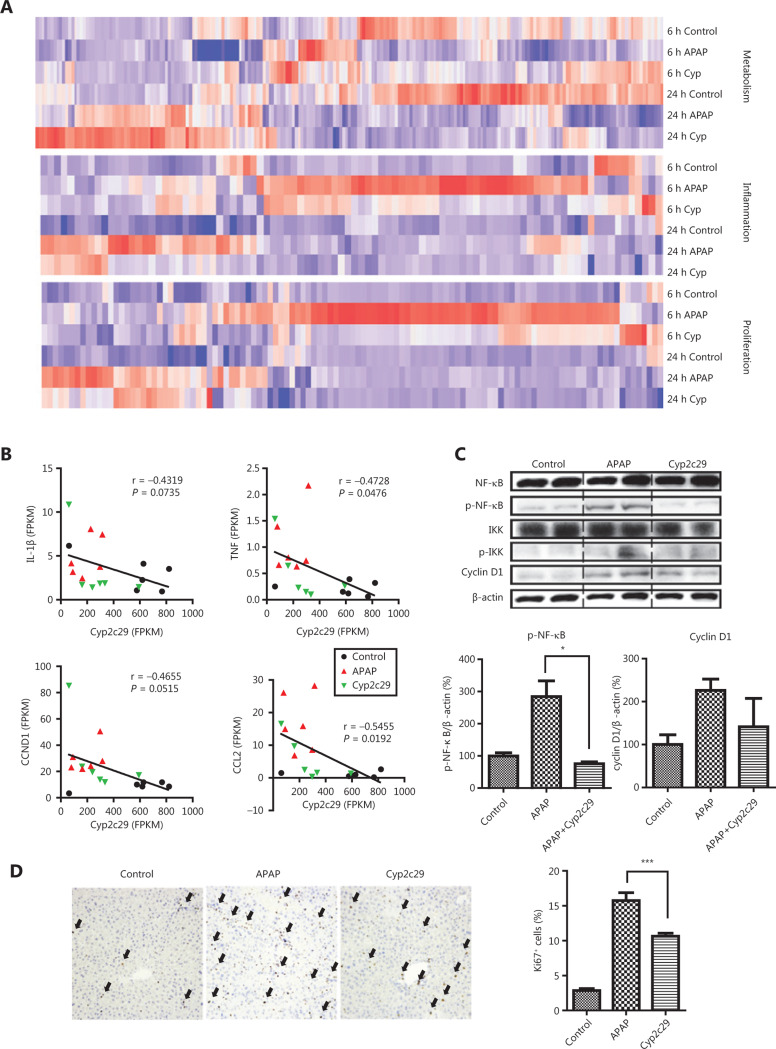
*Cyp2c29* attenuated NF-κB activation and inflammation in APAP-induced liver injury models. (A) Expression patterns of DEGs associated with metabolism, inflammation, and proliferation pathways. (B) Correlations between *Cyp2c29* expression and IL-1β, TNF, CCND1, and CCL2 expression. (C) Western blot analysis of p-NF-κB, p-IKK, and cyclin D1 expression in APAP-induced liver injury. (D) Immunohistochemical staining and counts of Ki67-positive cells. Black arrows: Ki67-positive cells. *P < 0.05, ***P < 0.001.

To further demonstrate the role of *Cyp2c29* in liver injury, we performed RNA-seq to detect gene expression in APAP models treated with *Cyp2c29* plasmid or empty vector plasmid for 6 h or 24 h. We identified 131 upregulated and 261 downregulated DEGs at 6 h, and 81 upregulated and 254 downregulated DEGs at 24 h, by comparing the *Cyp2c29* treated group with the APAP model group (**Supplementary Figure S8**). Next, we investigated the specific functions of the DEGs (**Figure 5A**). Compared with the control group (at 6 h and 24 h), the APAP model group showed suppressed metabolism and increased inflammation and cell death, whereas the opposite effects were observed for the APAP group treated with *Cyp2c29* plasmid. Cell differentiation and programmed cell death pathways were highly active in the APAP model group but suppressed in the *Cyp2c29*-treated group. These effects were more significant at 24 h than at 6 h after treatment (**Figure 5A**). We further investigated the TF regulation of the genes in 10 significant pathways. Eleven TFs were found to regulate genes associated with cell proliferation, inflammatory response, and metabolism pathways (**Figure 5B**). Furthermore, we explored the gene expression patterns of these pathways and found that liver tissues in the APAP model group exhibited high inflammatory activity and cell differentiation/death activity at 6 h and 24 h, whereas these activities were decreased in the *Cyp2c29*-treated group at 6 h and 24 h (**Figure 6A**). To further reveal the detailed mechanism, we mapped the DEGs between the *Cyp2c29*-treated group and APAP model group at 6 h to inflammation-related pathways, including the NF-κB, MAPK, TNF, and IL-17 pathways, and found that these pathways were all suppressed (**Supplementary Figure S9**).

Notably, the expression of Cyp2c29 was inversely correlated with that of IL-1β, TNF, CCND1, and CCL2 (**Figure 6B**), which are involved in the NF-κB pathway^43^. The protein levels of phosphorylated NF-κB p65 (also known as p-NF-κB p65 or p-NF-κB) were lower in the *Cyp2c29* group than in the APAP model group (**Figure 6C**). An upstream regulator of NF-κB, IKK, was phosphorylated and activated in the APAP model group; however, Cyp2c29 overexpression decreased the levels of phospho-IKK (**Figure 6C**). Therefore, Cyp2c29 suppressed the IKK-NF-κB signaling pathway. In the CCl_4_-induced mouse model, lower p-NF-κB was also observed in the *Cyp2c29* group than the CCl_4_ model group (**Supplementary Figure S7F**). These results indicated that the NF-κB pathway was suppressed in both the APAP- and CCl_4_-induced liver injury models after pre-treatment with the *Cyp2c29* plasmid. Immunohistochemical staining of Ki67 showed less liver cell proliferation in the *Cyp2c29* group than the APAP model group (**Figure 6D**), thus indicating less compensatory regeneration during liver injury. In agreement with these results, cyclin D1 was confirmed to be downregulated in the *Cyp2c29* group (**Figure 6C**).

### Expression of human homologs of mouse *Cyp2c29* is positively correlated with survival time in patients with HCC

**Figure 7:**
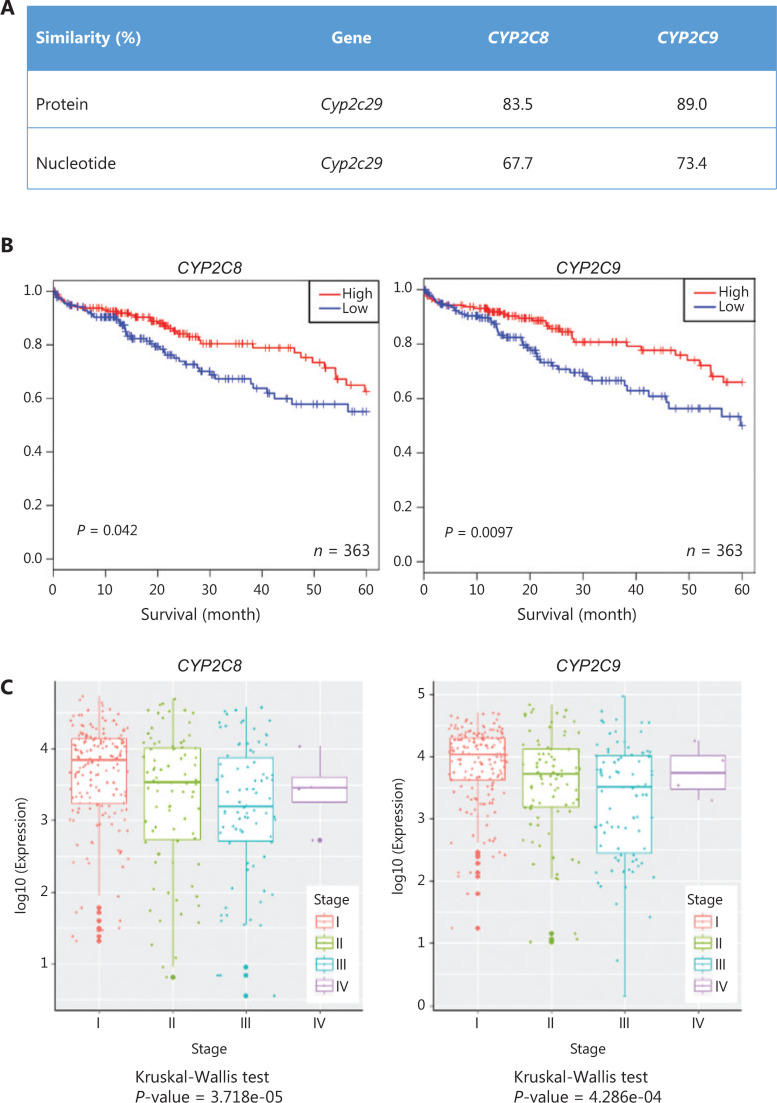
The expression of human homologs of mouse *Cyp2c29* is positively correlated with survival time in patients with HCC. (A) Similarities between *Cyp2c29* and human *CYP2C* epoxygenase genes. (B) Kaplan-Meier plots of 363 patients with HCC, stratified by *CYP2C8* and *CYP2C9* expression. Expression was classified as high or low on the basis of the median value. (C) Expression of *CYP2C8* and *CYP2C9* among patients with different cancer stages.

Among human epoxygenases, which are closely associated with lipid metabolism and EET production, the best-characterized members are CYP2C8, CYP2C9, and CYP2J2^44^. CYP2C8 and Cyp2c29 share 83.5% protein sequence similarity, whereas CYP2C9 and Cyp2c29 share 89.0% similarity (**Figure 7A**). To explore the association between CYP epoxygenase expression and clinical outcomes in patients with HCC, we performed an overall survival analysis, using gene expression data from 363 patients with HCC from TCGA. High expression of *CYP2C8* and *CYP2C9* was positively correlated with clinical survival (significance: *P* = 0.046 and 0.0097, respectively) (**Figure 7B**). Next, we quantified the gene expression levels of *CYP2C8* and *CYP2C9* in different HCC stages (TNM stages I, II, III, and IV) according to clinical information (**Figure 7C**). *CYP2C8* and *CYP2C9* expression significantly declined from stage I to stage III during HCC progression (Kruskal-Wallis, *P* < 0.001).

## Discussion

HCC, one of the most lethal cancers worldwide, occurs in the context of chronic liver injury and inflammation^45^. In our DEN-induced mouse model, we observed pathologic changes, including inflammatory infiltration, regeneration, fibrosis, and tumorous nodules, which are similar to changes associated with human HCC progression. On the basis of this model and time series gene expression analysis, we revealed *Cyp2c29* as a key gene in the progression from liver injury to HCC and further characterized its function.

*Cyp2c29* has been confirmed to be a specific CYP isoform responsible for EET-mediated vasodilator responses in mice^36^; however, its role in HCC progression remains largely unknown. We found that the proliferation of primary hepatocytes was enhanced by supernatant from LPS-activated RAW264.7 cells, whereas the effects were reversed by 14,15-EET treatment, in agreement with findings from a previous report of the anti-inflammatory activity of 14,15-EET^46–48^. Increases in EETs decrease vascular smooth muscle cell proliferation through downregulation of cyclin D1^49^. In agreement with these findings, our results showed that cyclin D1 expression was diminished while 14,15-EET was enhanced under conditions mimicking an inflammatory environment. Collectively, the production of EETs may partially explain why *Cyp2c29* suppresses compensatory proliferation during liver injury.

**Figure 8:**
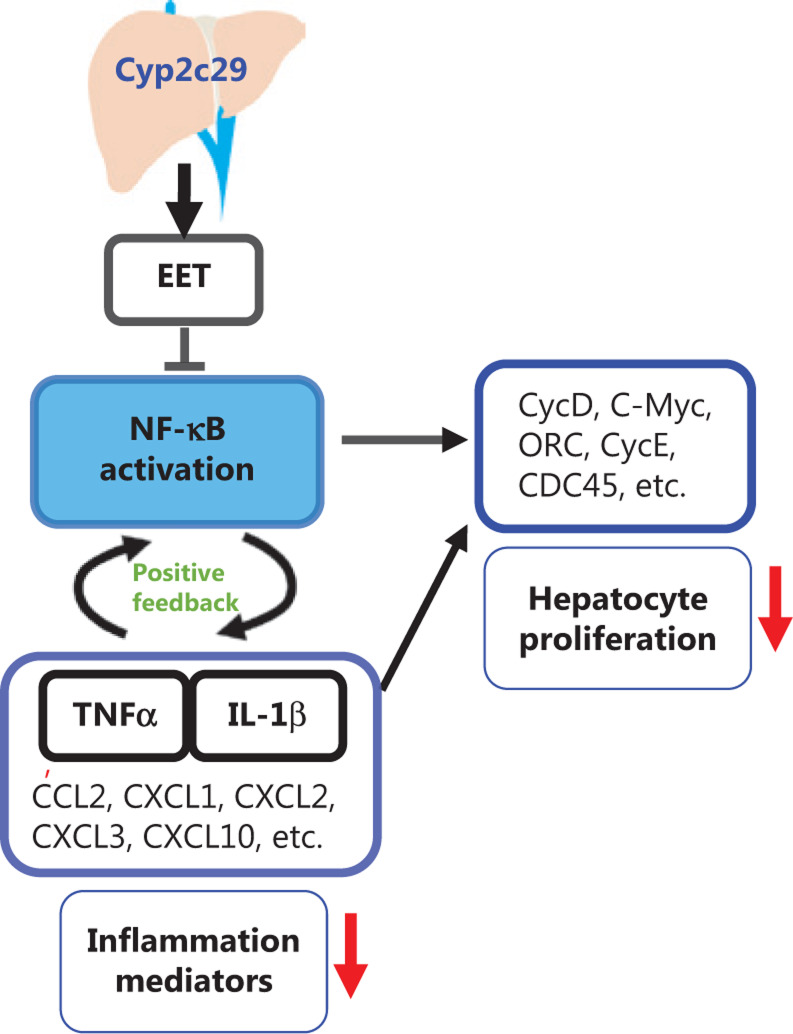
Pathway illustration. Overexpression of Cyp2c29 suppressed NF-κB activation by increasing the production of 14,15-epoxyeicosatrienoic acid (14,15-EET), which further inhibits the inflammatory and proliferation pathways. Downstream pathways of NF-κB, such as inflammatory cytokines (such as TNF and IL1), chemokines (such as CCL2, CXCL1, CXCL2, CXCL3, and CXCL10) and cell proliferation (such as CycD and CycE) were partly suppressed when Cyp2c29 was overexpressed in the mouse model of liver injury.

Inflammatory reactions are commonly correlated with HCC progression^50,51^. Our results showed that the NF-κB pathway was activated in the DEN-treated mouse model. APAP- and CCl_4_-induced liver injury induced an acute inflammatory response that was not similar to the DEN-induced inflammation in our study. However, APAP- and CCl_4_-induced liver injury models are characterized by typical inflammatory responses and NF-κB pathway activation^52,53^. Therefore, we used APAP- and CCl_4_-induced liver injury models to verify the role of *Cyp2c29.* Mice transfected with the* Cyp2c29* plasmid showed diminished injury and inflammation in both the APAP and CCl_4_ models, thus suggesting a protective effect of *Cyp2c29* against liver injury. When *Cyp2c29* was overexpressed, the levels of p-NF-κB and the expression of genes in NF-κB-related pathways, such as TNF and MAPK, which play important roles in inflammation and cell proliferation^54,55^, were downregulated. NF-κB is critical in the regulation of liver disease, influencing hepatocyte survival, and Kupffer cell and hepatic stellate cell activation^39^. NFκB activation is induced by infection, necrotic cell products, oxidative stress, and inflammatory cytokines such as TNF and IL-1^43^. Moreover, NF-κB activation mediates the synthesis of proinflammatory cytokines (such as TNF, IL1, and IL6) and chemokines (such as CXCL1, CXCL2, and CCL2)^56^. NF-κB and its related inflammatory molecules form a positive feedback loop and stimulate hepatocyte proliferation and liver regeneration, which may increase the risk of HCC. We found that *Cyp2c29* overexpression significantly downregulated NF-κB signaling. We performed network analysis and illustrated the involvement of *Cyp2c29* in signaling pathways (**Figure 8**). Genes that encode inflammatory cytokines (such as TNF and IL1), chemokines (such as CCL2, CXCL1, CXCL2, and CXCL3) and cell proliferation-related proteins (such as CycD and CycE) were downregulated when *Cyp2c29* was overexpressed in a mouse model of liver injury. These results demonstrate that Cyp2c29 may decrease liver injury through suppression of the inflammatory response.

High expression levels of *CYP2C8* and *CYP2C9*, which are highly homologous to *Cyc2C29*, were positively correlated with survival time in patients with HCC. Diminished expression levels of *CYP2C8* and *CYP2C9* were observed from stage I to stage III in patients with HCC. However, expression of another homologous gene, *CYP2J2*, was not significantly associated with survival time in patients with HCC in the TCGA data (**Supplementary Figures 10**), although CYP2J2 expression has been reported to be associated with tumor progression^57,58^. This finding suggests that the roles of CYP2J2 may vary in different cancers. These data together indicate the important roles of Cyp2c29 in liver injury and HCC progression.

In conclusion, Cyp2c29 was downregulated during liver injury in different mouse models. Cyp2c29 overexpression enhanced 14,15-EET production and suppressed inflammation-induced hepatocyte proliferation by inhibiting the IKK-NF-κB pathway during liver injury. However, whether the prolonged increase in expression of Cyp2c29 reduces inflammation-driven hepatocarcinogenesis remains unknown. Long term effects of Cyp2c29 must be further investigated. Our data provide evidence that CYP2C epoxygenases may be potential therapeutic targets for liver diseases. Future studies investigating the effects of other epoxygenases in the CYP2C family may elucidate additional regulators involved in HCC progression.

## References

[1] Ferlay J, Soerjomataram I, Dikshit R, Eser S, Mathers C, Rebelo M (2015). Cancer incidence and mortality worldwide: sources, methods and major patterns in GLOBOCAN2012. Int J Cancer..

[2] Colombo M, Maisonneuve P (2017). Controlling liver cancer mortality on a global scale: still a long way to go. J Hepatol.

[3]  He G, Karin M (2011). NF-kappa B and STAT3 – key players in liver inflammation and cancer. Cell Res.

[4]  Balkwill F, Mantovani A (2013). Inflammation and cancer: back to Virchow?. Lancet.

[5] Ray K (2018). A complex interplay between inflammation and immunity in liver cancer. Nat Rev Gastro Hepat.

[6]  Tacke F, Zimmermann HW (2014). Macrophage heterogeneity in liver injury and fibrosis. J Hepatol.

[7]  Li H, Clarke JD, Dzierlenga AL, Bear J, Goedken MJ, Cherrington NJ (2017). In vivo cytochrome P450 activity alterations in diabetic nonalcoholic steatohepatitis mice. J Biochem Mol Toxicol.

[8]  Yu D, Green B, Marrone A, Guo Y, Kadlubar S, Lin D (2015). Suppression of CYP2C9 by microRNA hsa-miR-128-3p in human liver cells and association with hepatocellular carcinoma. Sci Rep..

[9]  Nebert DW, Dalton TP (2006). The role of cytochrome P450 enzymes in endogenous signalling pathways and environmental carcinogenesis. Nat Rev Cancer.

[10]  Schuck RN, Zha W, Edin ML, Gruzdev A, Vendrov KC, Miller TM (2014). The Cytochrome P450 epoxygenase pathway regulates the hepatic inflammatory response in fatty liver disease. PLoS One.

[11]  Tsunedomi R, Iizuka N, Hamamoto Y, Uchimura S, Miyamoto T, Tamesa T (2005). Patterns of expression of cytochrome P450 genes in progression of hepatitis C virus-associated hepatocellular carcinoma. Int J Oncol..

[12]  Xu XR, Huang J, Xu ZG, Qian BZ, Zhu ZD, Yan Q (2001). Insight into hepatocellular carcinogenesis at transcriptome level by comparing gene expression profiles of hepatocellular carcinoma with those of corresponding noncancerous liver. Proc Natl Acad Sci USA.

[13]  Wang X, Yu T, Liao X, Yang C, Han C, Zhu G (2018). The prognostic value of CYP2C subfamily genes in hepatocellular carcinoma. Cancer Med..

[14]  Zhou L, Du Y, Kong L, Zhang X, Chen Q (2018). Identification of molecular target genes and key pathways in hepatocellular carcinoma by bioinformatics analysis. Oncotargets Ther.

[15]  Lin Y, Sibanda VL, Zhang HM, Hu H, Liu H, Guo AY (2015). MiRNA and TF co-regulatory network analysis for the pathology and recurrence of myocardial infarction. Sci Rep.

[16]  Lin Y, Zhang Q, Zhang HM, Liu W, Liu CJ, Li Q (2015). Transcription factor and miRNA co-regulatory network reveals shared and specific regulators in the development of B cell and T cell. Sci Rep..

[17]  Ye H, Liu X, Lv M, Wu Y, Kuang S, Gong J (2012). MicroRNA and transcription factor co-regulatory network analysis reveals miR-19 inhibits CYLD in T-cell acute lymphoblastic leukemia. Nucleic Acids Res..

[18]  Park EJ, Lee JH, Yu GY, He G, Ali SR, Holzer RG (2010). Dietary and genetic obesity promote liver inflammation and tumorigenesis by enhancing IL-6 and TNF expression. Cell.

[19]  Bakiri L, Wagner EF (2013). Mouse models for liver cancer. Mol Oncol.

[20]  Hoque R, Sohail MA, Salhanick S, Malik AF, Ghani A, Robson SC (2012). P2X7 receptor-mediated purinergic signaling promotes liver injury in acetaminophen hepatotoxicity in mice. Am J Physiol Gastrointest Liver Physiol..

[21]  Pertea M, Kim D, Pertea GM, Leek JT, Salzberg SL (2016). Transcript-level expression analysis of RNA-seq experiments with HISAT, StringTie and Ballgown. Nat Protoc..

[22]  Xia J, Sinelnikov IV, Han B, Wishart DS (2015). MetaboAnalyst 3.0 – making metabolomics more meaningful. Nucleic Acids Res..

[23]  Liu F, Song Y, Liu D (1999). Hydrodynamics-based transfection in animals by systemic administration of plasmid DNA. Gene Ther.

[24]  Shen GF, Jiang JG, Fu XN, Wang DW (2008). Promotive effects of epoxyeicosatrienoic acids (EETs) on proliferation of tumor cells. Ai Zheng.

[25]  Amaral SS, Oliveira AG, Marques PE, Quintão JL, Pires DA, Resende RR (2013). Altered responsiveness to extracellular ATP enhances acetaminophen hepatotoxicity. Cell Commun Signal..

[26]  Aparicio-Vergara M, Tencerova M, Morgantini C, Barreby E, Aouadi M (2017). Isolation of Kupffer cells and hepatocytes from a single mouse liver. Methods Mol Biol.

[27]  Aparicio-Vergara M, Tencerova M, Morgantini C, Barreby E, Aouadi M (2015). Isolation of non-parenchymal cells from the mouse liver. Methods Mol Biol.

[28]  Zhang HM, Kuang S, Xiong X, Gao T, Liu C, Guo AY (2015). Transcription factor and microRNA co-regulatory loops: important regulatory motifs in biological processes and diseases. Brief Bioinform.

[29]  Kockmann T, Trachsel C, Panse C, Wahlander A, Selevsek N, Grossmann J (2016). Targeted proteomics coming of age – SRM, PRM and DIA performance evaluated from a core facility perspective. Proteomics.

[30]  Uhlén M, Fagerberg L, Hallström BM, Lindskog C, Oksvold P, Mardinoglu A (2015). Proteomics. Tissue-based map of the human proteome. Science..

[31]  Liu CJ, Hu FF, Xia MX, Han L, Zhang Q, Guo AY (2018). GSCALite: a web server for gene set cancer analysis. Bioinformatics.

[32]  Polonikov A, Bykanova M, Ponomarenko I, Sirotina S, Bocharova A, Vagaytseva K (2017). The contribution of CYP2C gene subfamily involved in epoxygenase pathway of arachidonic acids metabolism to hypertension susceptibility in Russian population. Clin Exp Hypertens.

[33]  Ross AC, Zolfaghari R (2011). Cytochrome P450s in the regulation of cellular retinoic acid metabolism. Annu Rev Nutr.

[34] Danielson PB (2002). The cytochrome P450 superfamily: biochemistry, evolution and drug metabolism in humans. Curr Drug Metab.

[35]  Racanelli V, Rehermann B (2006). The liver as an immunological organ. Hepatology.

[36]  Sun D, Yang YM, Jiang H, Wu H, Ojaimi C, Kaley G (2010). Roles of CYP2C29 and RXR gamma in vascular EET synthesis of female mice. Am J Physiol Regu Integr Comp Physiol..

[37]  Tacconelli S, Patrignani P (2014). Inside epoxyeicosatrienoic acids and cardiovascular disease. Front Pharmacol.

[38]  Boron WF, Boulpaep EL (2002). Medical physiology: a cellular and molecular approach.

[39]  Luedde T, Schwabe RF (2011). NF-κB in the liver-linking injury, fibrosis and hepatocellular carcinoma. Nat Rev Gastro Hepat.

[40]  Tan H, He Q, Li R, Lei F, Lei X (2016). Trillin reduces liver chronic inflammation and fibrosis in carbon tetrachloride (CCl_4_) induced liver injury in mice. Immunol Invest.

[41]  Jiang L, Yue S, Li C, Ke M, Busuttil RW, Kupiec-Weglinski JW (2017). TAK1 signaling is essential for NLRP3 activation in mouse drug-induced damage-associated hepatitis. J Hepatol..

[42]  McGuinness PH, Painter D, Davies S, McCaughan GW (2000). Increases in intrahepatic CD68 positive cells, MAC387 positive cells, and proinflammatory cytokines (particularly interleukin 18) in chronic hepatitis C infection. Gut.

[43]  Taniguchi K, Karin M. (2018). NF-kappa B, inflammation, immunity and cancer: coming of age. Nat Rev Immunol..

[44]  Bishop-Bailey D, Thomson S, Askari A, Faulkner A, Wheeler-Jones C (2014). Lipid-metabolizing CYPs in the regulation and dysregulation of metabolism. Annu Rev Nutr.

[45] Bishayee A (2014). The role of inflammation and liver cancer. Adv Experimental Med Biol (Inflamm Cancer).

[46]  Theken KN, Deng Y, Kannon MA, Miller TM, Poloyac SM, Lee CR (2011). Activation of the acute inflammatory response alters cytochrome P450 expression and eicosanoid metabolism. Drug Metab Dispos.

[47]  Schmelzer KR, Kubala L, Newman JW, Kim IH, Eiserich JP, Hammock BD (2005). Soluble epoxide hydrolase is a therapeutic target for acute inflammation. Proc Natl Acad Sci USA.

[48]  Oni-Orisan A, Deng Y, Schuck RN, Theken KN, Edin ML, Lih FB (2013). Dual modulation of cyclooxygenase and CYP epoxygenase metabolism and acute vascular inflammation in mice. Prostaglandins Other Lipid Mediat..

[49]  Davis BB, Thompson DA, Howard LL, Morisseau C, Hammock BD, Weiss RH (2002). Inhibitors of soluble epoxide hydrolase attenuate vascular smooth muscle cell proliferation. Proc Natl Acad Sci USA.

[50]  Karin M, Lin A (2002). NF-kappa B at the crossroads of life and death. Nat Immunol.

[51]  Iimuro Y, Nishiura T, Hellerbrand C, Behrns KE, Schoonhoven R, Grisham JW (1998). NF kappa B prevents apoptosis and liver dysfunction during liver regeneration. J Clin Invest..

[52]  Ruepp SU, Tonge RP, Shaw J, Wallis N, Pognan F (2002). Genomics and proteomics analysis of acetaminophen toxicity in mouse liver. Toxicol Sci.

[53]  Son G, Iimuro Y, Seki E, Hirano T, Kaneda Y, Fujimoto J (2007). Selective inactivation of NF-kappa B in the liver using NF-kappa B decoy suppresses CCl_4_-induced liver injury and fibrosis. Am J Physiol Gastrointest Liver Physiol.

[54]  Wagner EF, Nebreda AR (2009). Signal integration by JNK and p38 MAPK pathways in cancer development. Nat Rev Cancer.

[55]  Grivennikov SI, Karin M (2011). Inflammatory cytokines in cancer: tumour necrosis factor and interleukin 6 take the stage. Ann Rheum Dis.

[56]  Tak PP, Firestein GS (2001). NF-kappaB: a key role in inflammatory diseases. J Clin Invest.

[57]  Jiang JG, Chen CL, Card JW, Yang S, Chen JX, Fu XN (2005). Cytochrome P450 2J2 promotes the neoplastic phenotype of carcinoma cells and is up-regulated in human tumors. Cancer Res..

[58] Panigrahy D, Kaipainen A, Greene ER, Huang S (2010). Cytochrome P450-derived eicosanoids: the neglected pathway in cancer. Cancer Metastasis Rev.

