# Supplementary material for: Time serial transcriptome reveals Cyp2c29 as a key gene in hepatocellular carcinoma development
Source: Cancer Biol Med. 2020 May 15;17(2):401–17. doi: 10.20892/j.issn.2095-3941.2019.0335 (PMC7309465; doi:10.20892/j.issn.2095-3941.2019.0335)
Supplement: Supplementary file 1 [file cbm-17-401-s001.pdf]

Supplementary materials

Table S1 Primer sequences for *GAPDH*, *Cyp2c29*, *IL-1*β and *TNF*-α

Primer	Sequence
GAPDH-S	GTTCCTACCCCCAATGTGTCC
GAPDH-A	TAGCCCAAGATGCCCTTCAGT
Cyp2c29-S	CAGATGTCACAGCTAAAGTC
Cyp2c29-A	TTTAATGTCACAGGTCACTG
IL-1β-S	TGCCACCTTTTGACAGTGATG
IL-1β-A	GCAGCCTTGTCCCTTGAAGA
TNF-α-S	CCCACGTCGTAGCAAACCAC
TNF-α-A	GCAGCCTTGTCCCTTGAAGA

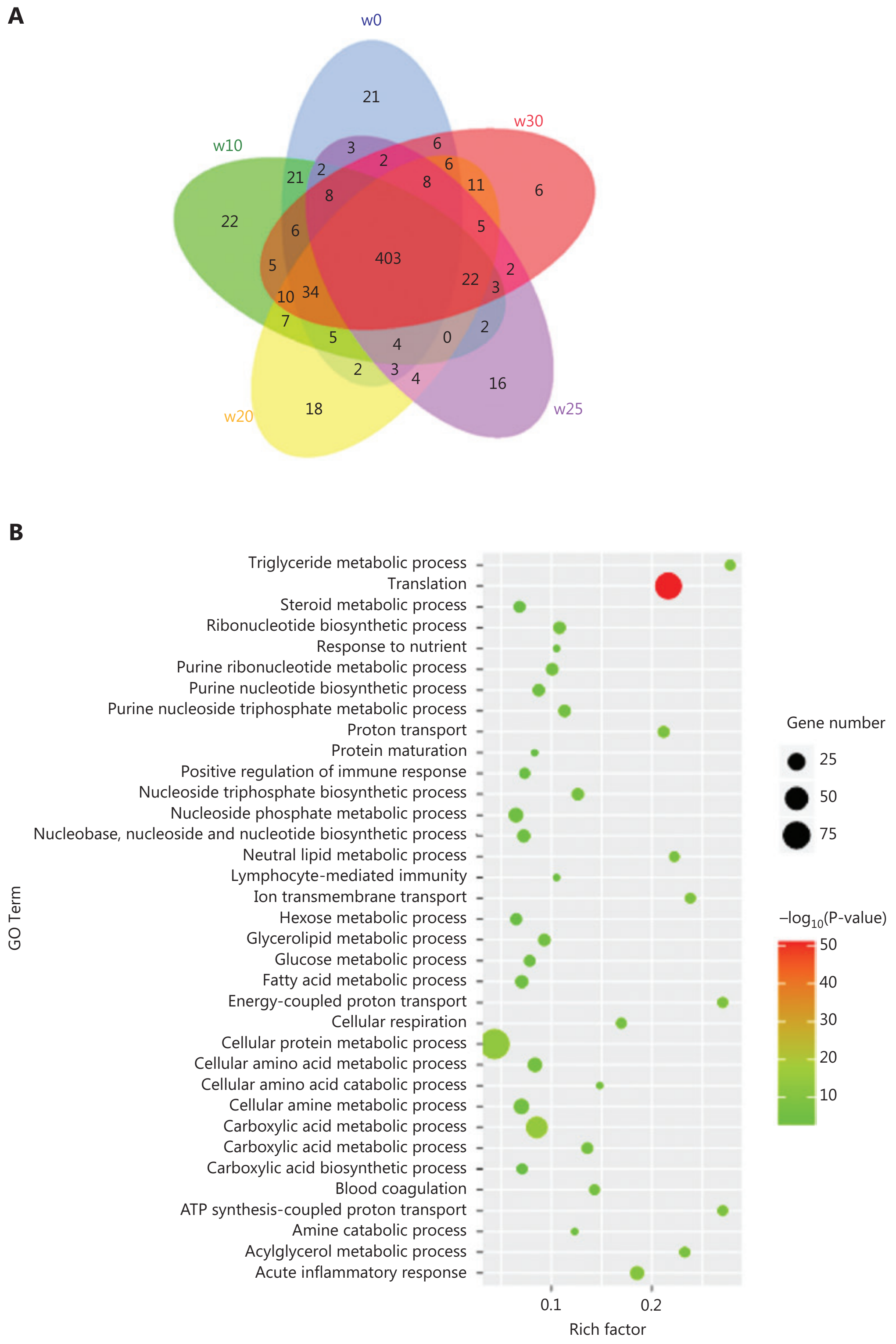

Figure S1 Overlap of highly expressed genes among the 5 groups. (A) Venn diagram of the highly expressed genes in the DEN model (FPKM > 80). (B) Functional enrichment for the highly expressed genes.

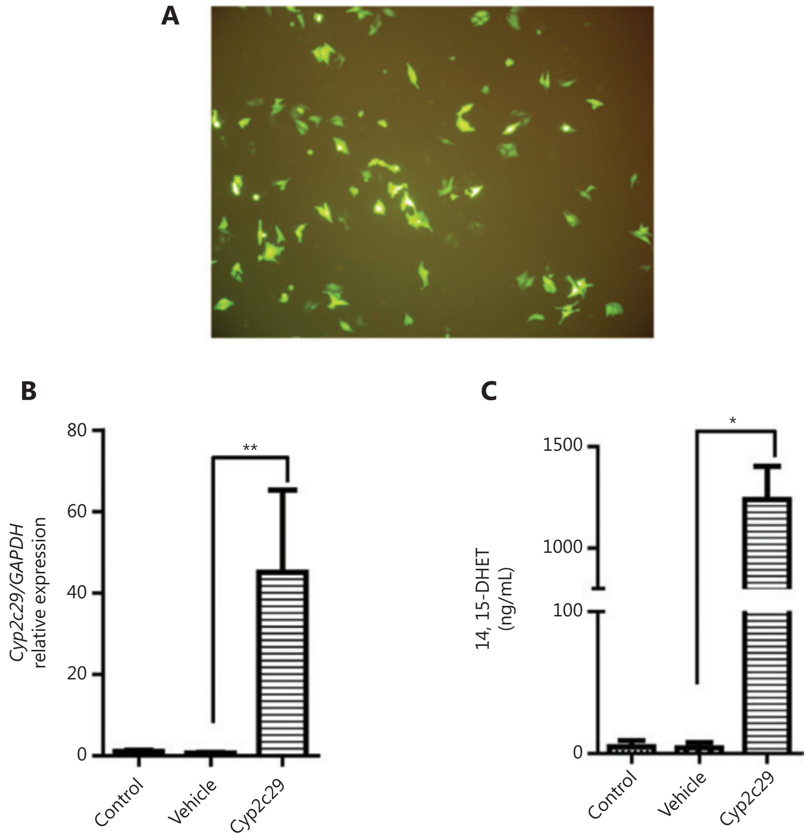

Figure S2 Cyp2c29 plasmids were successfully transfected into HL-7702 cells. (A) Image showing green fluorescence of HL-7702 cells transfected with Cyp2c29 pENTER-C-GFP plasmid. (B) qRT-PCR analysis of Cyp2c29 mRNA expression in Cyp2c29 plasmid-transfected HL-7702 cells. (C) ELISA of 14,15-DHET (14,15-EET hydrolysate) levels in the supernatant in Cyp2c29 plasmid-transfected HL-7702 cells. *P < 0.05; **P < 0.01.

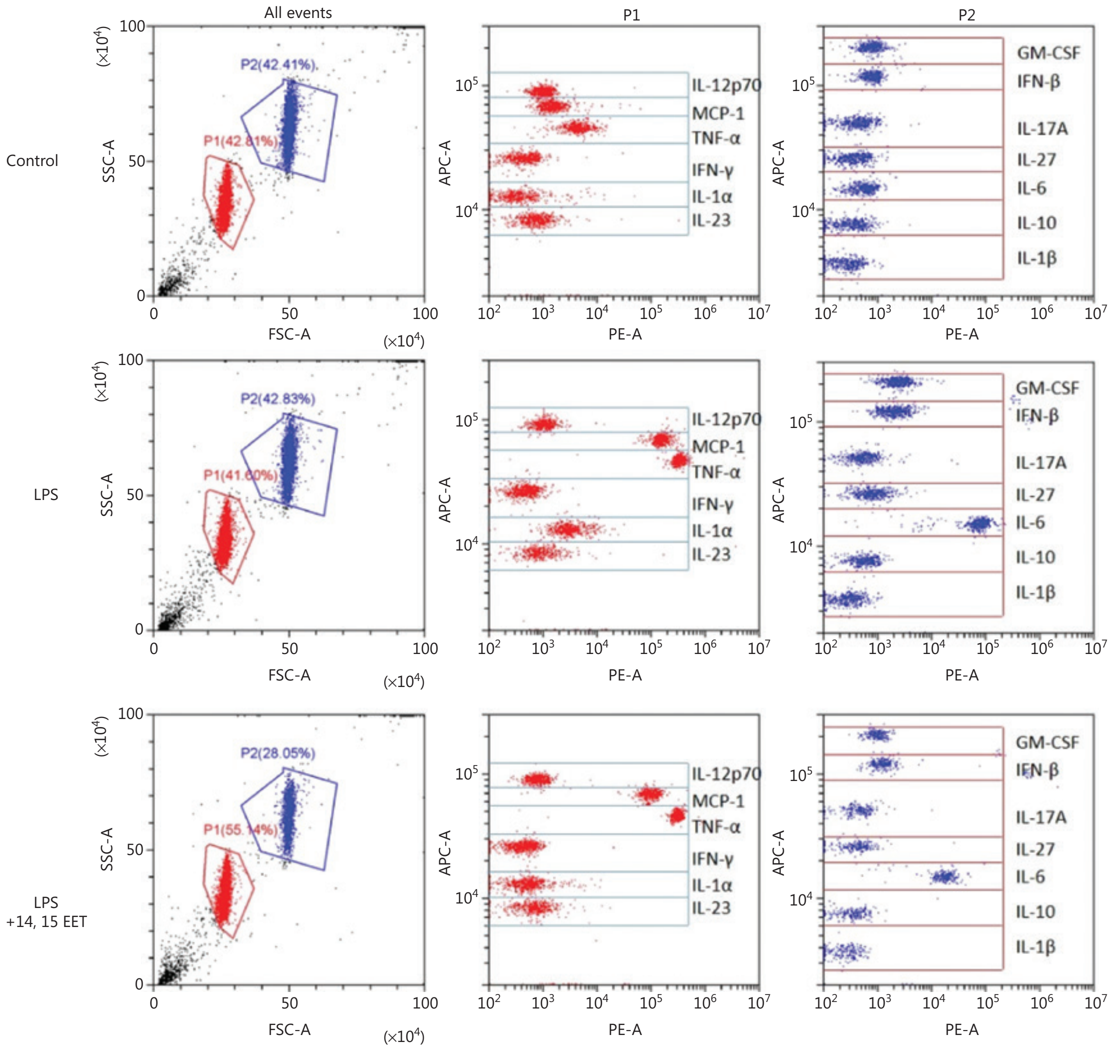

Figure S3 Gating of fluorescence-labeled beads for quantification of cytokines in the supernatant of the RAW264.7 cells.

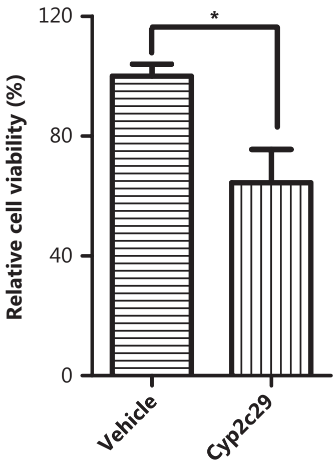

Figure S4 Viability of *Cyp2c29*-transfected H22 cells incubated with supernatant from LPS-activated RAW264.7 macrophages. *P < 0.05.

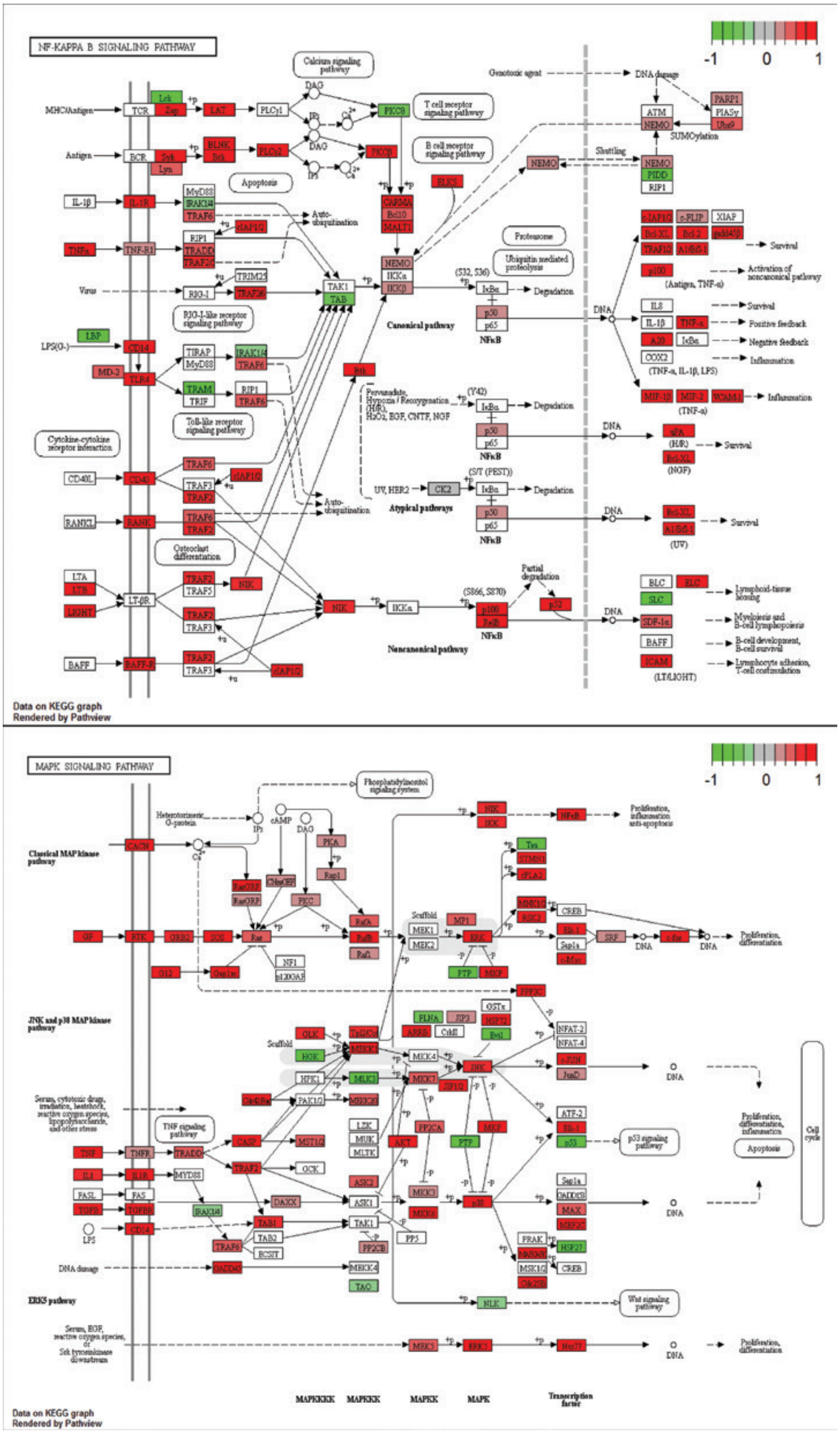

Figure S5 *NF-kB* pathway- and *MAPK* pathway-related genes differentially expressed in the DEN group *vs.* the control group in week 10. Green: downregulated gene; red: upregulated gene.

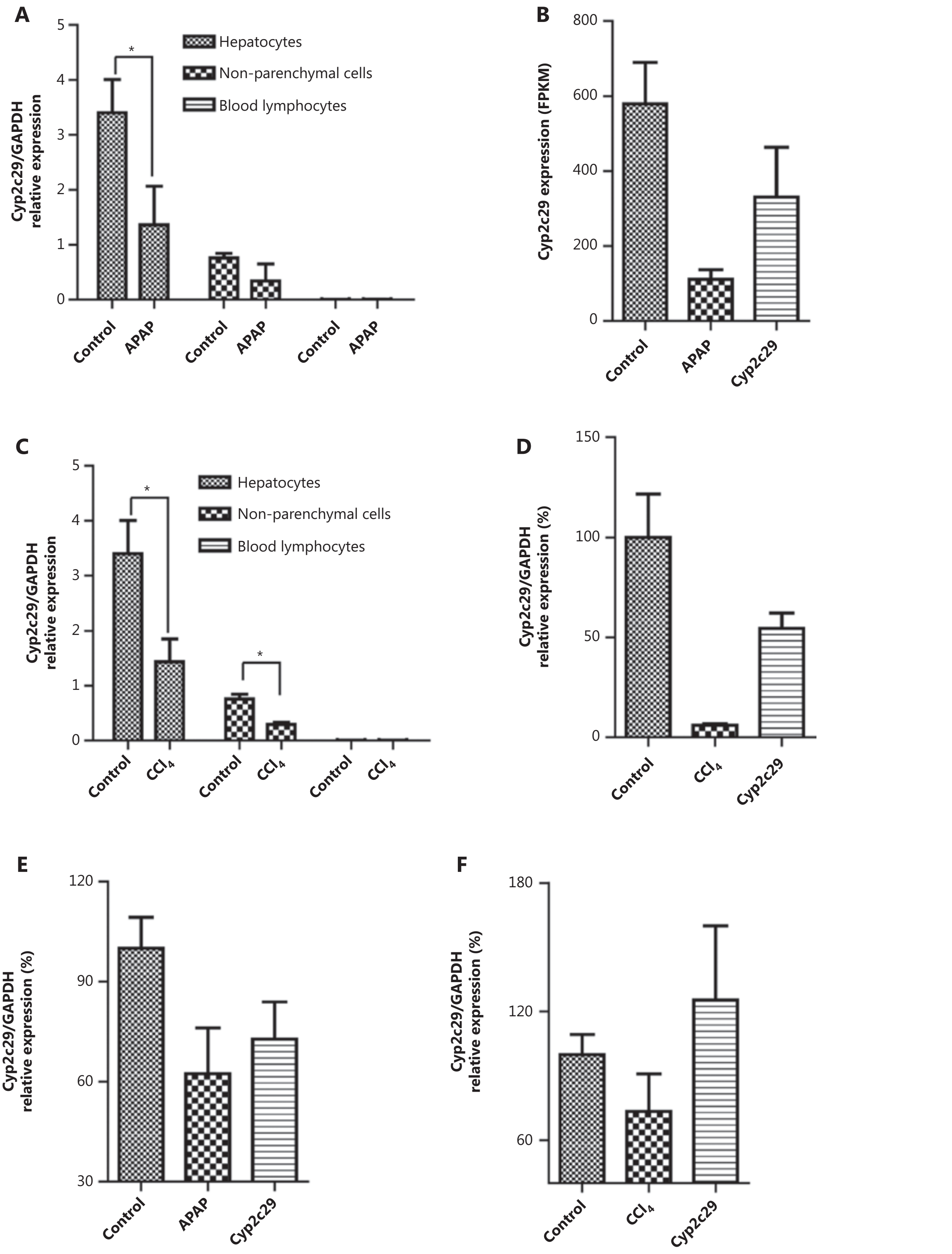

Figure S6 Expression levels of *Cyp2c29* after transfection. (A) *Cyp2c29* mRNA expression levels in different cells in the liver from APAP-treated mice after transfection. (B) *Cyp2c29* mRNA expression in liver tissue with injury induced by APAP after *Cyp2c29* plasmid transfection. (C) *Cyp2c29* mRNA expression in different cells in the liver from CCl_4-treated mice after transfection. (D) *Cyp2c29* mRNA expression in the injured liver tissue induced by CCl_4 after treatment with *Cyp2c29* plasmid transfection. (E) Level of Cyp2c29 protein in APAP-induced liver injury, as determined by targeted proteomics analysis. (F) Level of Cyp2c29 protein in CCl_4-induced liver injury, as determined by targeted proteomics analysis. *$P < 0.05$.

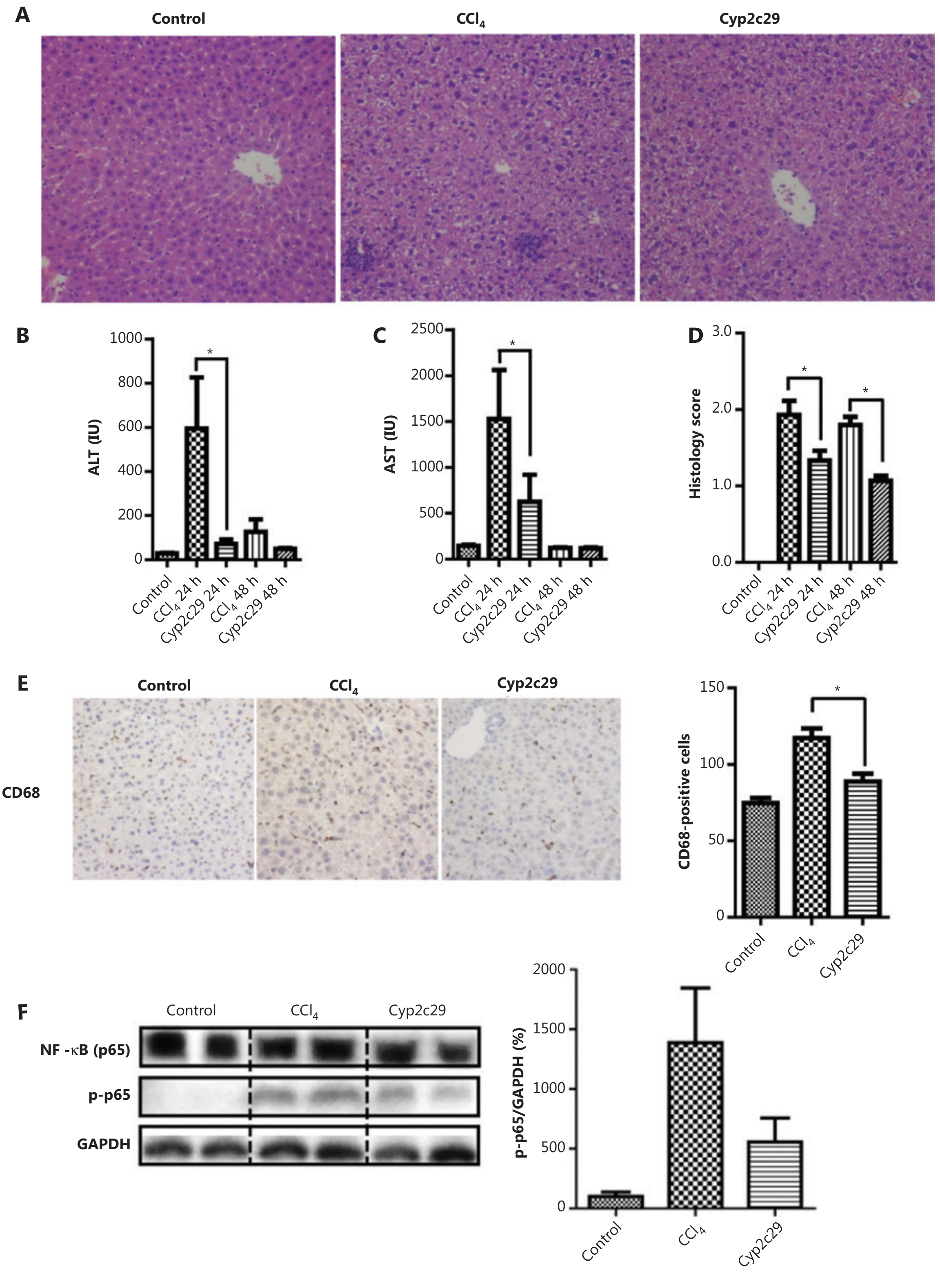

Figure S7 Overexpression of *Cyp2c29* decreased inflammation in the CCl_4-induced liver injury model. The mice in the *Cyp2c29* group received 100 μg of *Cyp2c29* plasmid DNA 48 h before CCl_4 administration, and the CCl_4 group received an equivalent empty vector 48 h before CCl_4 administration. The control group did not receive CCl_4 or plasmid DNA. (A) Liver H&E staining. (B) (C) ALT and AST levels at 24 h and 48 h after CCl_4 administration. (D) Histology score of APAP-induced liver injury. (E) Immunohistochemical staining of CD68-positive cells. (F) Western blot analysis of p-NF-κB (p-65) expression in CCl_4-induced liver injury. *$P < 0.05$.

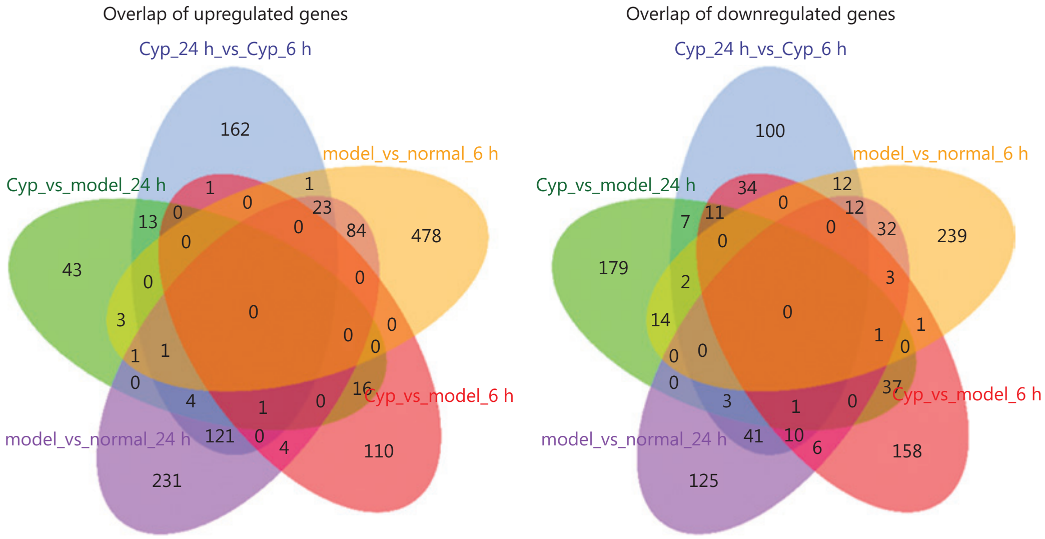

Figure S8 Identification of DEGs and overlap of up- and downregulated DEGs in the APAP model.

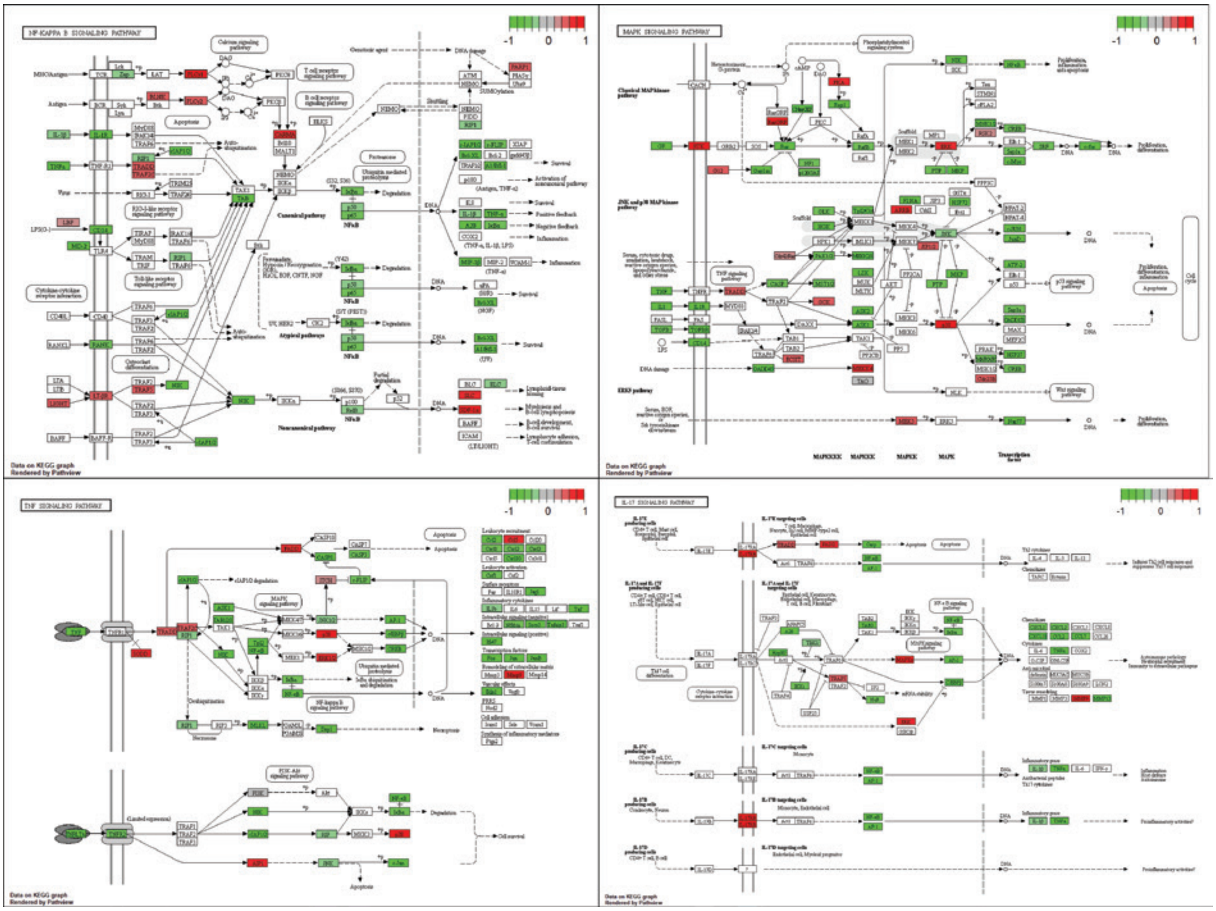

Figure S9 *NF-kB* pathway-, *MAPK* pathway-, *TNF* pathway-, and *IL-17* pathway-related DEGs in the *Cyp2c29* group *vs.* the APAP (model) group. Green: downregulated gene; red: upregulated gene.

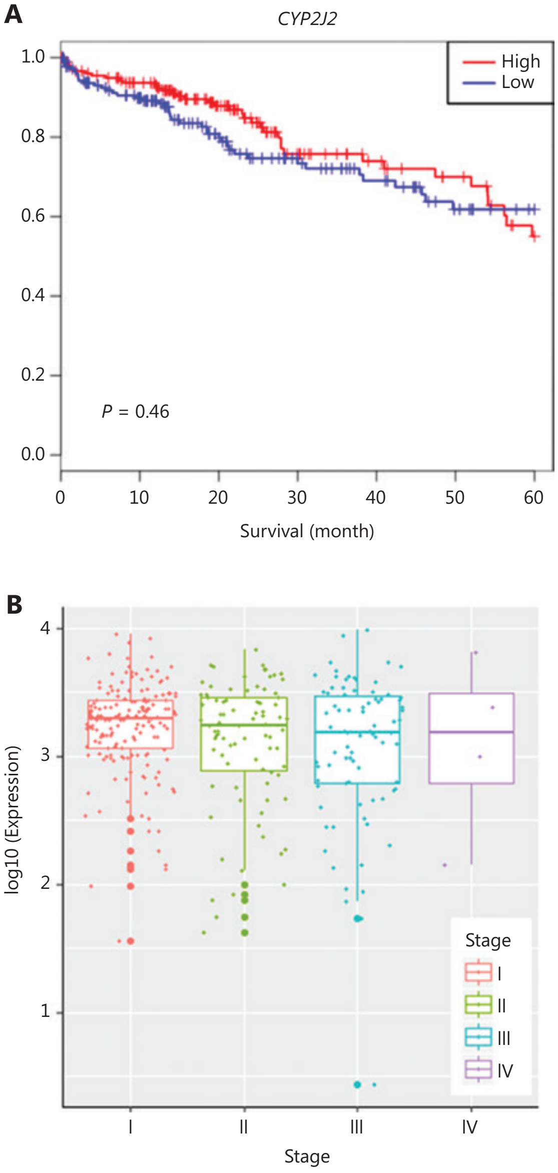

Figure S10 *CYP2J2* and survival time of patients with HCC. (A) Kaplan-Meier plots of 363 patients with HCC, stratified by *CYP2J2* expression. Expression was classified as high or low on the basis of the median value. (B) Expression of *CYP2J2* among patients with different HCC stages.
